# Novel MSVPWM to Reduce the Inductor Current Ripple for Z-Source Inverter in Electric Vehicle Applications

**DOI:** 10.1155/2014/586082

**Published:** 2014-04-16

**Authors:** Qianfan Zhang, Shuai Dong, Ping Xue, Chaowei Zhou, ShuKang Cheng

**Affiliations:** Institute of Electromagnetic and Electronic Technology, Harbin Institute of Technology, Harbin 150001, China

## Abstract

A novel modified space vector pulse width modulation (MSVPWM) strategy for Z-Source inverter is presented. By rearranging the position of shoot-through states, the frequency of inductor current ripple is kept constant. Compared with existing MSVPWM strategies, the proposed approach can reduce the maximum inductor current ripple. So the volume of Z-source network inductor can be designed smaller, which brings the beneficial effect on the miniaturization of the electric vehicle controller. Theoretical findings in the novel MSVPWM for Z-Source inverter have been verified by experiment results.

## 1. Introduction

**Figure 1:**
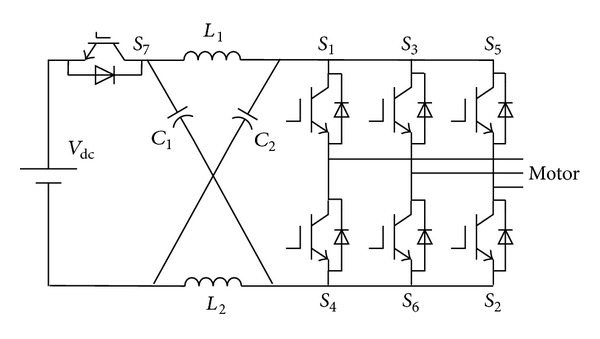
Bidirectional Z-Source inverter for electric vehicle.

Electric vehicles (EV) have attracted widespread research interest worldwide due to their attractive contributions to reducing gasoline consumption and carbon dioxide emissions [1]. DC bus voltage can be adjusted and the shoot-through state is allowed in Z-Source inverter (ZSI) [2], which improves the reliability of the inverter. By shifting between the shoot-through and non-shoot-through states, capacitors and inductors are charged and discharged, which boosts the DC-link voltage. Since it was proposed, Z-Source inverter is highly regarded by the researchers. Many research works have been done for electrical vehicle applications [3–6]. Connecting with bidirectional Z-Source network between DC power source and inverter (Figure 1), bidirectional Z-Source network can solve the mismatch problem of battery voltage and DC bus voltage, which ensures the DC bus voltage constant and enlarges working area of the electric machines.

However, the Z-Source inverter itself also has some inherent defects, such as the volume of inductor and capacitor taking up much space. Especially in electric vehicle applications, it affected the miniaturization of motor drives. In order to reduce the volume of Z-Source inductor, there are generally two kinds of measures. One is adopting coupled inductor [7, 8] and the other is improving modulation method to decrease the inductance current ripple.

Many PWM control methods have been proposed for ZSI by far, including simple SPWM [2], maximum SPWM [9], maximum constant boost control [10], and modified SVPWM (MSVPWM) [11, 12]. Compared with SPWM, MSVPWM has many advantages such as high voltage utilization, low current harmonics, and low switching losses [13], which is widely used in vector control and flux weakening control of electric vehicle motor drives.

**Figure 2:**
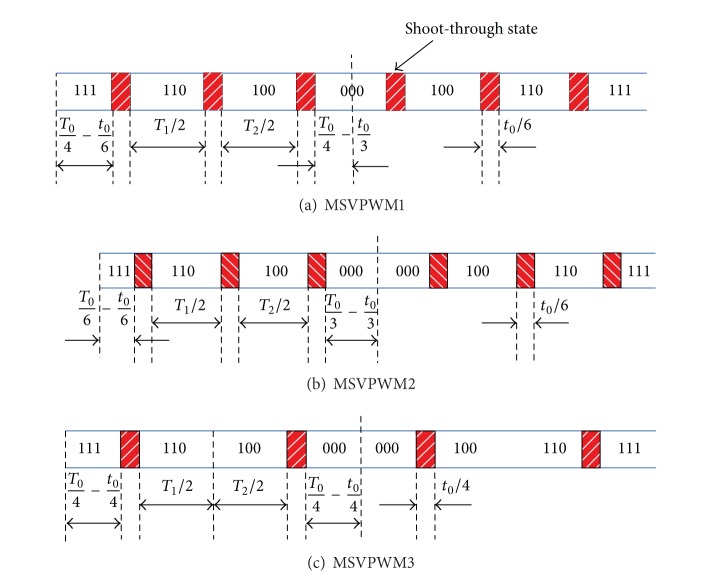
Vector distribution for existing MSVPWM.

There are three different MSVPWM to distribute the shoot-through states: MSVPWM1 [14, 15], MSVPWM2 [15, 16], and MSVPWM3 [17] (Figure 2). For MSVPWM1, the shoot-through state is evenly divided into six parts. This pattern succeeds in replacing the zero state with shoot-through state while keeping the switch frequency unchanged, but the maximum boost control cannot be achieved. The divided six-part shoot-through time intervals are also fulfilled in each control cycle in MSVPWM2, achieving maximum shoot-through time. MSVPWM3 divides the shoot-through state into four parts evenly and inserts them between adjacent two zero states and active state. Compared with MSVPWM1 and MSVPWM2, shoot-through state between two active states disappears.

In [18], the author obtained the conclusion that the MSVPWM1 and MSVPWM2 resulted in the lower current ripples when compared with the MSVPWM3. However, from the perspective of inductor design, we are more concerned about the maximum inductor current ripple, which directly affects the volume and weight of the inductor. In this paper, we draw a conclusion that the MSVPWM3 resulted in the lower maximum current ripples when compared with the MSVPWM1 and MSVPWM2. Next section describes the process of detailed analysis. In [19], the shoot-through time intervals of three phase legs are calculated and rearranged according to the active state and zero state time intervals to achieve the minimum current ripple across the Z-source inductor. In fact, with this modulation strategy, the maximum current ripple across Z-source inductor is equal to the MSVPWM3.

In this paper, we proposed a novel MSVPWM strategy, called MSVPWM4, which divides shoot-through state into six parts but fixes two states' position. It can achieve lower maximum inductor current ripple than the MSVPWM mentioned above.

## 2. Analysis of Maximum Current Ripple for Existing MSVPWM

**Figure 3:**
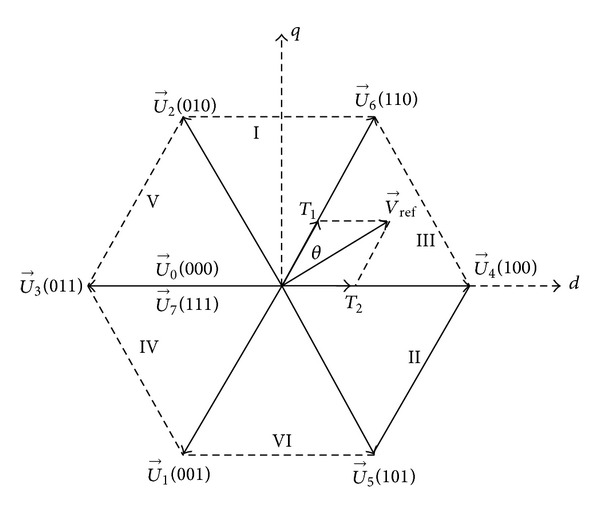
Space vector diagram.

Traditional SVPWM divides the space into six sectors with eight space vectors as shown in Figure 3, among which *U*_1_ ~ *U*_6_ are the six active vectors and *U*_0_, *U*_7_ are the two zero vectors. ${\overset{⃑}{V}}_{\text{ref}}$ is the reference vector. *T*_1_ and *T*_2_ are acting time of *U*_6_ and *U*_4_ in sector III with the expression as (1).  *T*_0_, *t*_0_, and *T*_*s*_ are the acting time of zero vector, shoot-through zero vector, and sampling period, respectively. *V*_dc_ is DC voltage and *m* is the modulation index.

Modified SVPWM applied in ZSI has additional shoot-through zero vector partly or fully replacing conventional zero vector without changing the active vector time. Consider

(1) T1=3|V⃑ref|VdcTssin(π3−θ)T2=3|V⃑ref|VdcTssinθ 0≤θ≤π3T0=Ts−T1−T2.

**Figure 4:**
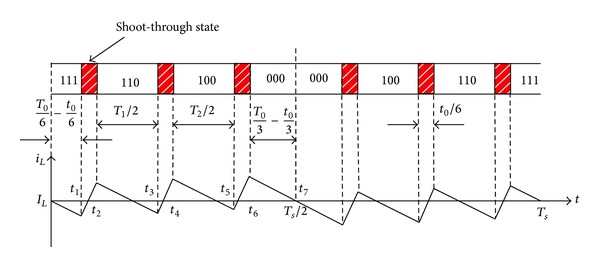
Inductor current ripple for MSVPWM2.

Assuming that the reference vector is in sector III, the distribution of vectors and the inductor current ripple for MSVPWM2 are shown in Figure 4. The maximum shoot-through time is increased to the zero vector time *T*_0_ accordingly and maximum boost control can be achieved.

For MSVPWM2, the inductor is charged during shoot-through period and inductor current rises, while the inductor discharges during non-shoot-through period and inductor current declines. The instantaneous values of inductor current are the same at the beginning and the end of a sampling period, both being equal to average inductor current *I*_*L*_. Because space vector PWM is symmetrical, the waveform of inductor current is centrosymmetric around the middle point of sampling period *T*_*s*_/2. So the current value at *T*_*s*_/2 is *I*_*L*_ too. The instantaneous inductor current ripple can be achieved as (2) [20]

(2) y1=Vin−VcLt1,y2=y1+VcL(t2−t1),y3=y2+Vin−VcL(t3−t2),y4=y3+VcL(t4−t3),y5=y4+Vin−VcL(t5−t4),y6=y5+VcL(t6−t5).

Inductor current ripple:

(3) $$\begin{array}{c} \Delta i_{L-2}=2\max\left(\left|y_{1}\right|,\left|y_{2}\right|,\left|y_{3}\right|,\left|y_{4}\right|,\left|y_{5}\right|,\left|y_{6}\right|\right). \end{array}$$

**Figure 5:**
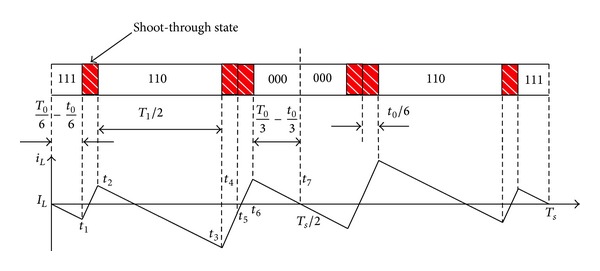
Inductor current ripple for MSVPWM2 with  *θ* = 0°.

The maximum inductor current ripple occurs in the adjacent sector position where *θ* = 0° or *θ* = 60°. On this working point, the current ripple is shown in Figure 5.

The maximum inductor current ripple for MSVPWM2 becomes

(4) $$\begin{array}{c} \Delta i_{L-2}=\frac{Dm}{2Lf_{s}\left(1-2D\right)}V_{\text{dc}}. \end{array}$$

With the similar analysis method above, the maximum inductor current ripple for MSVPWM1 and MSVPWM3 is

(5) $$\begin{array}{c} \Delta i_{L-1}=\frac{D\left(1+\left(3/4\right)m\right)}{2Lf_{s}\left(1-2D\right)}V_{\text{dc}}\\ \Delta i_{L-3}=\frac{\sqrt{3}Dm}{4Lf_{s}\left(1-2D\right)}V_{\text{dc}}. \end{array}$$

For MSVPWM1 and MSVPWM2, the time of two different active states varies, thus causing the shoot-through state between them to move correspondingly. The space vector has six sector critical positions where shoot-through time becomes four parts instead of six and inductor current ripple becomes the biggest. For MSVPWM3, shoot-through state between two active states disappears. Though the time of two active states varies, the sum of them merely changes a little. The position of shoot-through states beside these two active states is almost unchanged, keeping the frequency of inductor current ripple constant, and the maximum current ripple is reduced than that of MSVPWM1 and MSVPWM2.

## 3. Novel MSVPWM to Reduce the Volume of Inductor

**Figure 6:**
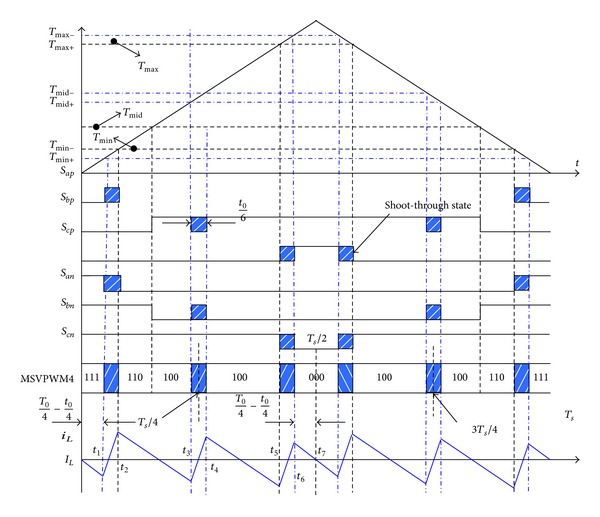
Vector distribution and inductor current ripple for MSVPWM4.

To compromise the maximum current ripple and average current ripple, MSVPWM4 is presented. Based on MSVPWM3, MSVPWM4 divides shoot-through state into six parts again but fixes two states' position (Figure 6). From left to right, the second and fifth shoot-through state are fixed to (*T*_*s*_/4 − *t*_0_/12 ~ *T*_*s*_/4 + *t*_0_/12) and (3*T*_*s*_/4 − *t*_0_/12 ~ 3*T*_*s*_/4 + *t*_0_/12) separately. The frequency of inductor current ripple is six times the sampling frequency. It can achieve lower inductor current ripple. The disadvantage is increasing the switching frequency.

For MSVPWM4, maximum inductor current ripple occurs when (*T*1 + *T*2) reaches maximum when  *θ* = 30°. Inductor current is centrosymmetric within half a sampling period. The current values at *T*_*s*_/4, *T*_*s*_/2, and *T*_*s*_ are all equal to the average inductor current. Maximum inductor current ripple for MSVPWM4 can be calculated as

(6) $$\begin{array}{c} \Delta i_{L-4}=\frac{D\left(\left(\left(3\sqrt{3}\right)/2\right)m+D-1\right)}{6Lf_{s}\left(1-2D\right)}V_{\text{dc}}. \end{array}$$

**Figure 7:**
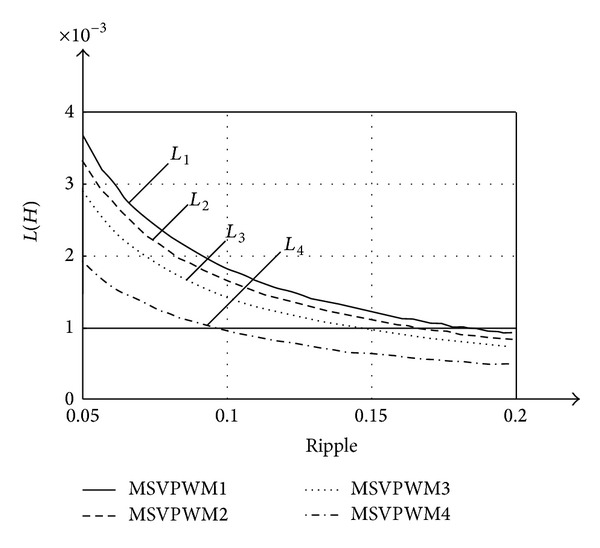
Inductance for different MSVPWM versus allowable inductor current ripple.

Compared with (4)–(6), we can conclude that Δ*i*_*L*−4_ < Δ*i*_*L*−3_ < Δ*i*_*L*−2_ < Δ*i*_*L*−1_.  Figure 7 shows the required inductance with different MSVPWM, using the same parameter of Δ*i*_*L*_, *V*_*dc*_, *f*_*s*_, *m*, and *D*. Obviously, the ZSI inductance designed with MSVPWM4 is much smaller than that with the other three MSVPWM, which is about 40% smaller than MSVPWM2.

## 4. Experimental Verification

**Figure 8:**
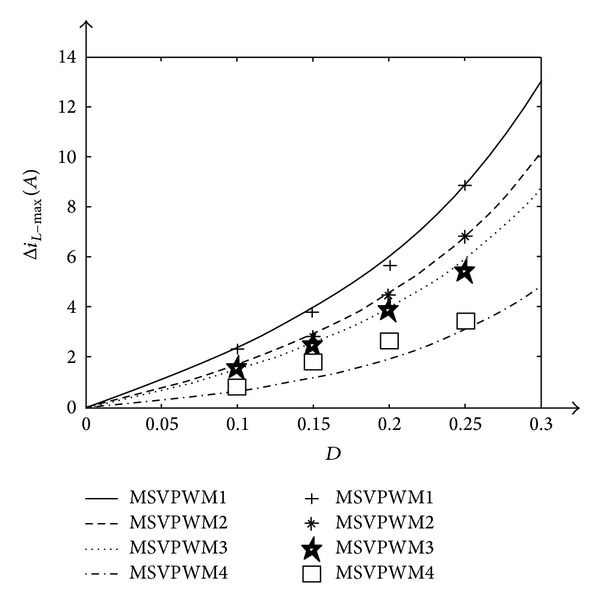
Comparison between theoretical predictions and experimental results of  Δ*i*_*L*_.

For illustration of the validity of the proposed approach, the maximum inductor current ripple is both theoretically calculated and experimentally measured for different MSVPWM under the conditions as Table 1. Experimental results are summarized in Table 2 and Figure 8.

**Figure 9:**
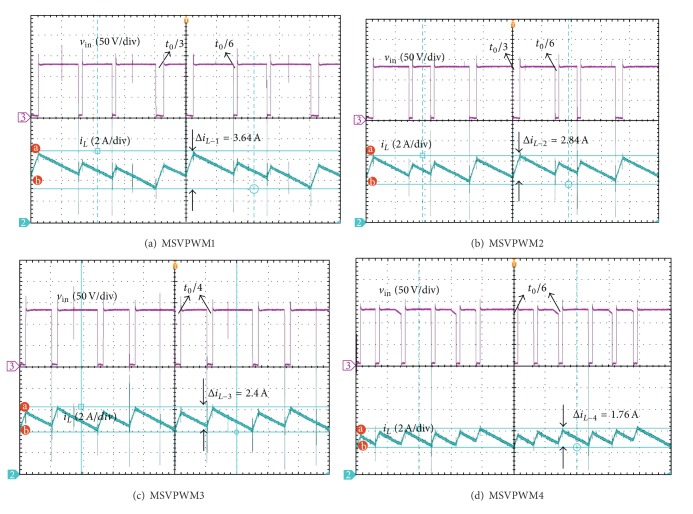
Maximum inductor current ripple for different MSVPWM with *D* = 0.15.

**Figure 10:**
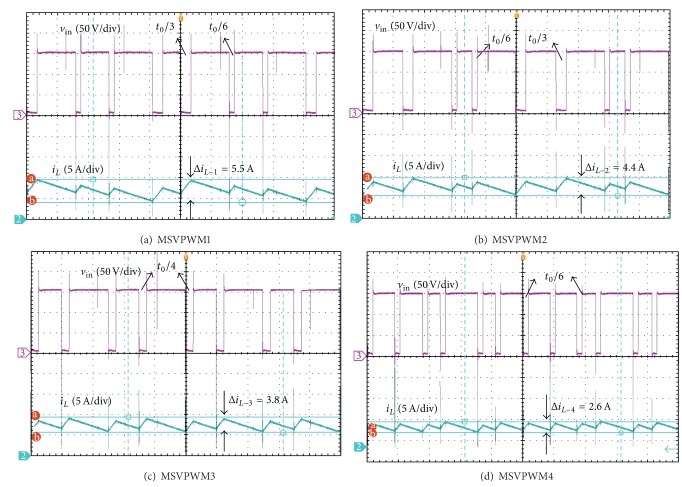
Maximum inductor current ripple for different MSVPWM with *D* = 0.2.

From Table 2 and Figure 8, it is noted that the proposed MSVPWM4 has the most superior performance in lowering maximum inductor current ripple when compared with other MSVPWM. The experiment results are very close to the theoretical analysis. Experiment results with *D* = 0.15 and *D* = 0.2 are shown in detail in Figures 9 and 10.

The experiment results show that DC link voltage is boost to 150V with *V*_dc_ = 90V when *D* = 0.2. The maximum inductor current ripple with proposed MSVPWM, called MSVPWM4, is 67.7% smaller than that of MSVPWM1, 57.8% smaller than MSVPPWM2, and 51.3% smaller than MSVPWM3.

## 5. Conclusions

In this paper, a novel MSVPWM with lower inductor current ripple for Z-Source inverter is proposed based on rearranging the distribution of shoot-through states. Experimental results are provided to verify the effectiveness of the proposed modulation method. The MSVPWM4 has smaller inductor current ripple than that of existing methods, thus reducing the volume of ZSI inductance, which brings the beneficial effect on the miniaturization of the drives on electric vehicles.

## Figures and Tables

**Table 1:** Parameters of experiment.

DC Source (Vdc)	Modulation Index (m)	Carrier Frequency (fs)	Z-Source inductor (L)	Z-Source capacitor (C)
90 v	0.6	2 kHz	1 mH	2700 uF

**Table 2:** Comparison between theoretical predictions and experimental results of Δ*i*_*L*_.

Δ*i*_*L*_(*A*)	MSVPWM1	MSVPWM2	MSVPWM3	MSVPWM4
*D* = 0.1				
Theoretical Predictions	2.20	1.69	1.46	0.62
Experimental Results	2.10	1.60	1.50	0.80
*D* = 0.15				
Theoretical Predictions	3.78	2.90	2.50	1.14
Experimental Results	3.64	2.84	2.40	1.76
*D* = 0.2				
Theoretical Predictions	5.87	4.50	3.91	1.90
Experimental Results	5.50	4.40	3.81	2.60
*D* = 0.25				
Theoretical Predictions	8.81	6.75	5.85	3.00
Experimental Results	8.80	6.80	5.42	3.40

## References

[1] Yu Y, Zhang Q, Liang B, Liu X, Cui S (2011). Analysis of a single-phase Z-Source inverter for battery discharging in vehicle to grid applications. *Energies*.

[2] Peng FZ (2003). Z-source inverter. *IEEE Transactions on Industry Applications*.

[3] Ellabban O, Van Mierlo J, Lataire P Control of a bidirectional Z-source inverter for hybrid electric vehicles in motoring, regenerative braking and grid interface operations.

[4] Ellabban O, van Mierlo J, Lataire P, Van Den Bossche P Z-source inverter for vehicular applications.

[5] Peng FZ, Shen M, Holland K (2007). Application of Z-source inverter for traction drive of fuel cell-battery hybrid electric vehicles. *IEEE Transactions on Power Electronics*.

[6] Shen M, Joseph A, Wang J, Peng FZ, Adams DJ (2007). Comparison of traditional inverters and Z-source inverter for fuel cell vehicles. *IEEE Transactions on Power Electronics*.

[7] Qin L, Peng FZ, Miaosen S Switched-coupled-inductor inverter.

[8] Yang S, Lei Q, Peng FZ, Inoshita R, Qian Z Current-fed quasi-Z-source inverter with coupled inductors.

[9] Peng FZ, Shen M, Qian Z Maximum boost control of the Z-source inverter.

[10] Shen M, Wang J, Joseph A, Peng FZ, Tolbert LM, Adams DJ (2006). Constant boost control of the Z-source inverter to minimize current ripple and voltage stress. *IEEE Transactions on Industry Applications*.

[11] Loh PC, Vilathgamuwa DM, Lai YS, Chua GT, Li Y (2005). Pulse-width modulation of Z-source inverters. *IEEE Transactions on Power Electronics*.

[12] Chun TW, Tran QV, Ahn JR, Lai JS AC output voltage control with minimization of voltage stress across devices in the Z-source inverter using modified SVPWM.

[13] Ali US, Kamaraj V A modified space vector PWM for bi-directional Z-source inverter.

[14] Jung JW, Keyhani A (2007). Control of a fuel cell based Z-source converter. *IEEE Transactions on Energy Conversion*.

[15] Ellabban O, van Mierlo J, Lataire P Comparison between different PWM control methods for different Z-source inverter topologies.

[16] Yushan L, Baoming G, Abu-Rub H, Fang Zheng P (2013). Control system design of battery-assisted quasi-Z-source inverter for grid-tie photovoltaic power generation. *IEEE Transactions on Sustainable Energy*.

[17] Ali US, Kamaraj V A novel space vector PWM for Z-source inverter.

[18] Yushan L, Baoming G, Abu-Rub H, Fang Zheng P (2014). Overview of space vector modulations for three-phase Z-source/quasi-Z-source inverters. *IEEE Transactions on Power Electronics*.

[19] Yu T, Shaojun X, Jiudong D (2014). Pulsewidth modulation of Z-source inverters with minimum inductor current ripple. *IEEE Transactions on Industrial Electronics*.

[20] Ding J, Xie S, Tang Y Optimal design of the inductor in Z-source inverter with single phase shoot-through SVPWM strategy.

